# Violence in Sudan: A Looming Public Health Disaster

**DOI:** 10.7759/cureus.40343

**Published:** 2023-06-12

**Authors:** Abdulqadir J Nashwan, Sarah H Osman, Leena A Mohamedahmed

**Affiliations:** 1 Department of Nursing, Hamad Medical Corporation, Doha, QAT; 2 Faculty of Medicine, University of Khartoum, Khartoum, SDN; 3 Department of Public Health, International University of Africa, Khartoum, SDN

**Keywords:** coordinated response, humanitarian crisis, national health laboratory seizure, health services disruption, sudan conflict

## Abstract

Sudan's ongoing conflict, rooted in colonial-era policies and resource competition, has led to widespread displacement, poverty, and social service breakdowns. The escalating power struggle between the Sudanese Army Forces (SAF) and the paramilitary Rapid Support Forces (RSF) exacerbates the humanitarian crisis by severely undermining the nation's health services and infrastructure. This leads to long-lasting social and economic consequences, creating a need for a coordinated response from national and international organizations to provide emergency healthcare, rebuild infrastructure, and train and retain healthcare workers. Moreover, the recent takeover of a National Public Health Laboratory in Khartoum, which contains dangerous biological material, is considered extremely dangerous. The expulsion of technicians and power cuts prevent the proper management of biological materials (e.g., polio, measles, and cholera isolates). This editorial sheds light on the deep-seated repercussions of the conflict in Sudan, with a specific focus on the toll it takes on health services and infrastructure. It calls for an all-encompassing, synergistic approach that places the health and welfare of impacted communities at the forefront. Through concerted collaboration between national entities and the global community, there lies the potential to pave the way for recuperation, fortitude, and enduring stability in regions ravaged by conflict.

## Editorial

The ongoing conflict in Sudan has caused significant disruption to the country's health services [1]. The conflict has been rooted for several years and involves multiple armed factions competing for dominance [2]. Nonetheless, a tumultuous surge of armed confrontations violently unfurled on April 15, 2023, marking a significant escalation in hostilities. Between April 15th and the writing of this paper, Sudan witnessed an alarming tally of 865 fatalities and more than 5,424 injuries, painting a grim picture of the human toll inflicted by the conflict [3]. The unsettling unrest has also ignited a massive upheaval in population movements, with an estimated 1.4 million individuals forced to abandon their homes [3]. Around one million remain within Sudan’s borders as internally displaced persons, while an additional 330,000 have sought sanctuary across neighboring nations' frontiers [3]. As a result of the conflict, health facilities have been damaged or destroyed, and medical personnel have been forced to flee. This has led to a significant impact on the health of the population, with many people struggling to access basic medical care. Additionally, the recent National Public Health Laboratory takeover has created new challenges, such as biological risks, as the laboratory contains polio, measles, and cholera isolates [4]. In this editorial, we will provide an overview of the conflict in Sudan, analyze the repercussions of this conflict on healthcare services, and explore the implications of the laboratory takeover and its potential consequences.

The Sudan conflict is a complex and long-lasting issue that has plagued the country for decades [5]. It has its roots in colonial-era policies that favored certain ethnic groups over others, leading to a legacy of inequality and resentment. Oil competition has also fuelled the conflict, leading to violent clashes between different groups (such as the Sudanese Armed Forces (SAF), paramilitary Rapid Support Forces (RSF), and the Southern Sudan People’s Liberation Army (SPLA)) [6]. Over the years, Sudan has undergone several major peace processes [7]. The Darfur Peace Process, initiated with the N'Djamena talks (2003-2004), culminated in the Darfur Peace Agreement in 2006. The Eastern Peace Process focused on Eastern Sudan with the Asmara talks in 2006, leading to the Eastern Sudan Peace Agreement. The North-South Peace Process, spanning decades, included the Addis Ababa Agreement (1972) and the Khartoum Peace Agreement (1997), and culminated in the Comprehensive Peace Agreement (2005), addressing power sharing, wealth distribution, and security. Additionally, the National Democratic Alliance (NDA) Government Talks resulted in the Cairo Agreement in 2005 [7]. According to Ameresekere and Henderson (2012), the conflict has displaced millions of people, widespread poverty, and a breakdown of social services [8]. The resulting humanitarian crisis has been exacerbated by the government's refusal to allow international aid organizations to operate freely in the country. Despite efforts by the international community to broker a lasting peace, the situation remains volatile, with sporadic outbreaks of violence continuing to occur [9]. The Sudan conflict is a stark reminder of the devastating impact that long-standing ethnic and resource-based conflicts can have on a country and its people. The current conflict in Sudan is primarily a power struggle between the SAF and the RSF [10]. Fighting for control has led to hundreds of deaths and a worsening humanitarian crisis. The RSF, a powerful militia group outside the regular armed forces' command, has allowed its leaders to control a large part of the economy, especially the gold trade [11]. As the struggle escalates, the ceasefire has given way to ground fighting, intensifying the conflict and its consequences for the African nation.

The impact of conflict on health services is a critical issue that affects the health and well-being of individuals and communities [11]. According to the doctors' syndicate in Sudan, as of May 2023, medical services have been halted in over 70% of hospitals in conflict-affected regions of the country, with 13 hospitals being targeted by bombings and 19 others being forcibly evacuated [12]. The ongoing conflict creates significant challenges for health services, including the disruption of healthcare delivery, the destruction of health infrastructure, and the displacement of healthcare workers [12]. These challenges often lead to decreased quality and accessibility of healthcare services. In addition, conflict can exacerbate existing health problems and create new health risks, such as the spread of infectious diseases due to poor sanitation and hygiene and increased mental health problems due to trauma and stress [13]. The negative impact of conflict on health services can be long-lasting and have significant social and economic consequences [1]. The challenges faced by health services in conflict-affected areas require a coordinated response from national and international organizations, including providing emergency healthcare services, rebuilding health infrastructure, and training and retaining healthcare workers.

United Nations (UN) officials reported that a faction in Sudan's conflict had taken control of the National Public Health Laboratory in Khartoum, holding dangerous biological material, marking an "extremely dangerous" development [1,4]. Amid a ceasefire, concerns grow about more refugees fleeing the chaos-stricken nation. The WHO warned of the "huge biological risk" associated with the lab's occupation [14]. With technicians expelled and power cuts, properly managing the lab's biological materials is impossible. In addition, the ongoing fighting and limited supplies have forced many hospitals to close, prompting the UN to warn of a potential health system collapse. The takeover of facilities housing biohazardous materials can usher in grave ramifications, particularly in the crucible of conflict, where the potential for nefarious use or unintended mishandling is heightened [15]. When armed groups take control of such facilities, they may compromise the safety and security of the stored materials, putting the surrounding population at risk. In addition, the expulsion of trained technicians and potential infrastructure damage can hinder proper management of the lab's contents, increasing the likelihood of accidental or deliberate release of dangerous pathogens. Within the context of an expansive conflict, the takeover of laboratory/research facilities can amplify pre-existing public health hurdles, give rise to novel hazards, and intensify the pressure on healthcare systems that are already treading on thin ice. Such actions can critically compromise the ability to respond to health emergencies and sustain essential services, adding layers of complexity to an already fraught situation.

In conclusion, conflict in Sudan exerts a substantial and far-reaching influence on health services, disrupting healthcare delivery, infrastructure, and workforce, and the recent takeover of the National Public Health Laboratory will only add to their struggles. To effectively address these challenges, a holistic and coordinated strategy must be implemented, prioritizing the health and well-being of affected communities. This approach should involve national and international organizations working collaboratively to provide emergency healthcare services, rebuild damaged infrastructure, and train and retain healthcare workers. Ultimately, focusing on the health needs of conflict-affected populations can promote recovery, resilience, and long-term stability in regions marred by strife.
